# Switching from cetrorelix to dydrogesterone in an IVF cycle - a new strategy for unexpected freeze-all cycles

**DOI:** 10.5935/1518-0557.20230036

**Published:** 2023

**Authors:** Roberto de Azevedo Antunes, Maria do Carmo Borges de Souza, Marcelo de Souza Marinho, Verônica de Almeida Raupp, Gabriela Palhano Sifuentes Melo

**Affiliations:** 1 Fertipraxis - Human Reproduction Center, Rio de Janeiro, Brazil; 2 Gynecology Department of the Clementino Fraga Filho Hospital from the Federal University of Rio de Janeiro, Brazil

**Keywords:** Assisted Reproductive Techniques, Ovulation Detection, Ovarian Hyperstimulation Syndrome, Progestagens, Progestins effects

## Abstract

Preventing a luteinizing hormone (LH) surge is a major concern in controlled ovarian stimulation (COS). Several strategies have been developed over the years, including protocols with Gonadotrophin Releasing Hormone agonists and antagonists. More recently Progestin Primmed Ovarian Stimulation (PPOS) has shown to be equally effective in pituitary suppression, with comparable clinical and laboratorial outcomes. This is the case of a 34 year old female, with a previous diagnosis of primary infertility due to tubal factor and high ovarian reserve markers. The initial plan was to perform IVF/ICSI. followed by fresh blastocyst transfer. The chosen COS strategy was to use Alfacorifolitropin 150mg (Elonva^®^) and Cetrorelix acetate 0,25mg (Cetrotide^®^) in a flexible pituitary suppression protocol. However, because of elevated risk for Ovarian Hyper-stimulation Syndrome (OHSS) detected during ultrasound and hormonal monitoring, in order to diminish financial burden and to have a more patient friendly protocol, we switched cetrorelix acetate to oral dydrogesterone. COS was successful and resulted in 24 retrieved oocytes (16 metaphase 2 oocytes) without any premature LH peak. No OHSS symptoms occurred. Our main goal with this case report is to reinforce the feasibility and efficacy of this innovative approach, especially in patients aiming for a fresh embryo transfer, who present alert sings of OHSS during the stimulation. Developing friendlier and cheaper protocols in assisted reproduction makes the treatment more accessible and affordable.

## INTRODUCTION

The occurrence of premature luteinizing hormone (LH) peaks in ovarian stimulation protocols has always been a concern in reproductive medicine. In the early days, up to 29% of the controlled ovarian stimulation cycles had to be canceled due to premature LH peaks that resulted in ovulation before oocyte retrieval (Eibschitz *et al*., 1986).

Several pituitary suppression strategies have been developed over the years, firstly involving the use of Gonadotrophin Releasing Hormone (GnRH) agonists, and later GnRH antagonists. Both are similarly effective in avoiding premature LH peaks, with a success rate ranging from 98-99%, and with no differences in clinical and laboratorial outcomes (Lambalk *et al*., 2017). From 2020 onwards, the European Society of Human Reproduction and Embryology (ESHRE) recommends GnRH antagonists as the main choice of suppressive pituitary therapy in IVF/ICSI cycles because it allows the use of GnRH agonist triggers to prevent Ovarian Hyperstimulation Syndrome (OHSS) (The ESHRE Guideline Group On Ovarian Stimulation, 2020).

The use of progestins as a tool to avoid premature LH peak in IVF cycles is more recent. It was first reported in 2015 in a comparison between oral medroxyprogesterone acetate (MPA) and a short GnRH agonist protocol. In this publication, no differences were reported regarding premature LH peak, cycle cancelation, or number of retrieved oocytes (Kuang *et al*., 2015).

After that, several progestin-primed ovarian stimulation (PPOS) protocols have been described, comparing MPA and GnRH antagonists (Beguería *et al*., 2019; Yildiz *et al*., 2019), Dydrogesterone and GnRH agonists (Yu *et al*., 2018), Dydrogesterone and GnRH antagonist (Iwami *et al*., 2018), oral micronized progesterone and GnRH agonists (Zhu *et al*., 2015). All showed equivalent pituitary suppression and similar clinical and laboratorial outcomes.

In PPOS protocols, progestin is usually started alongside gonadotropins. Because of the luteinizing effect these protocols have on the endometrium, they require that all embryos be frozen and transferred in a further cycle. They also allow the use of GnRH agonist triggers to effectively prevent OHSS (La Marca & Capuzzo, 2019).

A comparison of PPOS versus GnRH antagonist protocols shows a clear cost reduction in Ovarian Stimulation cycles scheduled not to involve fresh embryo transfers, or in social oocyte freezing cycles (Evans *et al*., 2019).

Ever since, PPOS protocols became a viable alternative to GnRH antagonists in patients at risk for OHSS, social oocyte cryopreservation cycles, preimplantation genetic analysis cycles, and any type of cycle that does not involve a fresh embryo transfer.

In 2020, a new approach to pituitary suppression was proposed. In this publication, patients with high ovarian reserve markers underwent ovarian stimulation for IVF/ICSI in an attempt to accomplish a fresh embryo transfer, and, therefore, used GnRH antagonist for suppression. However, during stimulation, because of elevated risk for OHSS (more than 13 follicles >11mm diameter), the authors changed the GnRH antagonists to MPA in order to diminish costs (MPA is cheaper than GnRH antagonist) and to make the cycle more patient friendly (MPA is given orally, while GnRH antagonists subcutaneously). There were no differences in premature LH peak, OHSS rates or severity or on clinical outcomes measured (Huang *et al*., 2020).

The present case report used the same principle of switching an GnRH antagonist for progestin in patient at high risk for OHSS who wished to try a fresh embryo transfer before stimulation. However, instead of oral MPA, we decided to use oral dydrogesterone. So far, this is the first case of a switch protocol described where a GnRH antagonist (Cetrorelix acetate 0.25mg) was substituted with dydrogesterone in the same stimulation cycle.

## CASE DESCRIPTION

M.C.O., aged 34 years, married, Body Mass Index (BMI) 22.71kg/m^2^, with a history of controlled systemic arterial hypertension, normal physiological development, and regular menses, had a medical history of laparoscopic myomectomy in January 2021, which had been interrupted due to intense bleeding associated with hemodynamic instability. In April 2021, a new myomectomy, this time via laparotomy, was performed without further complications. In September 2021, she discontinued the use of an oral contraceptive (Adoless^®^), which she had been using for the last nine years, and started trying to conceive. After nine months of unsuccessful attempts, she sought care at Fertipraxis Human Reproduction Center on June 8, 2022.

Her partner, F.A.F.S., aged 42 years, BMI 33.71kg/m^2^, had a history of mumps during and no other noteworthy event in his medical history. An automated spermogram from November 2022 showed no major alterations in the main seminal parameters.

Hysterosalpingography was performed in January 2022, which showed a coiled fallopian tube on the right, with a positive Cotte test, in addition to hydrosalpinx on the left. In May 2022, magnetic resonance imaging (MRI) of the pelvis revealed post-surgical alterations, multiple uterine fibroids without uterine cavity distortion and hydrosalpinx on the left. She also underwent a video hysteroscopy in May 2022, which confirmed no signs of uterine cavity distortion or endometritis. Her workup and serology tests were normal; anti-Müllerian hormone (AMH) was 2.27ng/mL in November 2022.

From the very beginning, the couple expressed concerns about the cost of treatment. Therefore, we went for controlled ovarian stimulation (COS) followed by a fresh blastocyst transfer. Due to her age, preimplantation genetic analysis (PGT-A) was not performed. However, before going through with COS, she was submitted to a new laparoscopy and a left salpingectomy to resolve the hydrosalpinx in August 2022.

**Figure 1:**
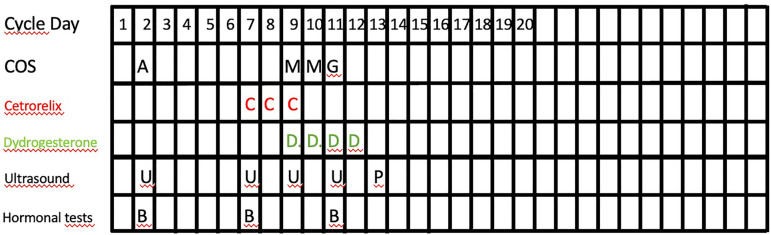
Schematics of the controlled ovarian stimulation.

On the cycle before COS, luteal phase priming was administered with oral norethisterone acetate 10mg (Primolut Nor^®^, Schering - Brazil), once a day. COS started on December 26, 2022, on day two of the follicular phase, with corifollitropin alfa 150mcg (Elonva^®^, Schering-Plough, Germany), single dose, subcutaneously (SC). The baseline antral follicle count was 24. Hormone levels on day 2 were as follows: estradiol (E2) 15pg/mL; FSH 3.47UI/mL; LH 4.00UI/mL; progesterone (P4) 0.15ng/mL. Cetrorelix acetate, 0.25mg (Cetrotide^®^, Merck, Germany) once a day, SC, was started on cycle day 7. At this time, the patient did not present clinical symptoms of OHSS. Ultrasound examination showed the largest follicle measuring 14mm, a total of 14 follicles between 9 to 14mm, five follicles < 9mm, and no free fluid in the posterior cul-de-sac. Hormone levels on this day were as follows: E2 1477pg/mL, LH 5.77UI/mL. Two days later, on day 9 of the cycle, ultrasound examination showed four follicles ≥ 16 mm, 14 follicles between 12 and 16mm, and a small amount of free fluid in the posterior cul-de-sac. At this time, the patient complained of slight pelvic discomfort, with no impact on her daily activities. As predicted, we started gonadotropin supplementation with 150UI of menotropin (Merional^®^, UCB, Switzerland) and decided to contraindicate the fresh embryo transfer considering the high risk of OHSS. Therefore, we switched to a pituitary suppression strategy and started supplementation with oral dydrogesterone (Duphaston^®^, Abbott, Holland) 10mg 3x a day. From day 10 onwards, cetrorelix acetate was discontinued. Ovulation trigger was performed on day 11 of the cycle, with 0.2mg of triptorelin acetate (Gonapeptyl daily^®^, Ferring, Germany), SC. Ultrasound examination performed on this day found 15 follicles ≥ 16mm and about the same amount of free fluid occupying the posterior cul-de-sac. Symptoms remained unchanged from the previous clinical evaluation. Hormone levels were as follows: E2 4992,0pg/mL, P4 1.25ng/mL, and LH 0.4UI/mL. Oocyte pick-up (OPU) was performed 36 h after LH trigger without complications. The patient was discharged with prophylactic measures against OHSS and a prescription of cabergoline 0.5mg a day (Dostinex^®^, Eurofarma, Brazil). Figure 1 shows the schematics of the controlled ovarian stimulation.

A - Corifollitropin alfa 150µg

M- Menotropin 150UI

G - Triptorelin Acetate 0.2mg

C - Cetrorelix Acetate 0.25mg

D- Dydrogesterone 30mg

U- ultrasound assessment

P - oocyte pick-up

B - hormonal blood assessment

Four days after OPU, the patient was clinically asymptomatic, and her ultrasound examination showed a right ovary with an estimated volume of 30.02mL and a left ovary with 82.74mL. Free fluid was again detected in the posterior cul-de-sac.

Ultrasound examination performed ten days after OPU showed normal ovarian volumes and no more free fluid in the posterior cul-de-sac.

Regarding laboratorial outcomes, a total of 24 oocytes, including 16 MII oocytes were identified. The fertilization rate was 50% after ICSI, despite normal parameters in the semen sample. Embryo culture resulted in three blastocysts (day 5 5BB, day 5 5BB, day 6 4BB).

## DISCUSSION

PPOS can effectively block the LH surge when started together with gonadotropins for ovarian stimulation (Guan *et al*., 2021). Most publications on PPOS cycles present with a follicular phase ovarian stimulation, with progestin usually started on the first day of ovarian stimulation (Kuang *et al*., 2015; Beguería *et al*., 2019; Yildiz *et al*., 2019; Yu *et al*., 2018; Iwami *et al*., 2018; Zhu *et al*., 2015).

Three meta-analyses showed that PPOS does not affect outcomes such as number of retrieved oocytes, number of retrieved mature oocytes, number of good quality embryos, implantation rate, clinical pregnancy rate, or live birth rate (Guan *et al*., 2021; Cui *et al*., 2021; Alexandru *et al*., 2020).

No differences in embryo euploidy rates were reported in a recent publication comparing the PPOS protocol and other GnRH agonist and antagonist protocols (Wang *et al*., 2023). When obstetrical and neonatal outcomes were analyzed, no differences were detected comparing several PPOS protocols with either GnRH agonists or antagonists cycles (Huang *et al*., 2019).

More recently, flexible start PPOS protocols have been proposed using MPA. They showed similar efficacy in blocking LH surges as GnRH antagonist protocols. In this same study, flexible PPOS protocols yielded a higher post trigger LH surge and higher mean number of mature oocytes (Kalafat *et al*., 2022).

The publication from Huang *et al.* (2020) is the only so far to propose the switch from GnRH antagonists to MPA in the same cycle. The group switched to MPA had, as expected, fewer days of GnRH antagonist administration, presented the same effect in suppressing premature LH surges, as well as the same clinical pregnancy and live birth rates. OHSS occurrence was the same in both groups (Huang *et al*., 2020). The present case report was inspired by the above-mentioned publication; however, we opted for a different switch strategy: we used dydrogesterone instead of MPA, which is unprecedented in the literature.

Our results show that the LH surge was effectively blocked even when Cetrorelix acetate was replaced with dydrogesterone. Good clinical outcomes were also reported, with 16 metaphase II oocytes retrieved out of 17 retrieved oocytes, with three good quality blastocysts frozen. No symptoms of OHSS symptoms were identified. Although this is a single case, its results show the viability and efficacy of this new approach when dydrogesterone is used as part of the pituitary suppression strategy in an ovarian stimulation cycle.

Both in the present case, as well as in Huang *et al.* (2020), the switch from an antagonist protocol to PPOS was performed for patients who would undergo fresh embryo transfers, and later had to change to a freeze-all approach due to risk of OHSS during stimulation. As a result, a more friendly and less costly IVF/ICSI cycle was offered. In countries such as Brazil, where most IVF centers are private and there are no government support policies for IVF, the ability to offer more affordable strategies must be pursued.

Dydrogesterone was chosen in our case firstly because of its properties in preventing the LH surge (Yu *et al*., 2018; Iwami *et al.,* 2018). Good clinical, obstetrical and neonatal outcomes have also been published in PPOS with dydrogesterone (Cui *et al*., 2021; Wang *et al*., 2023; Huang *et al*., 2019). Secondly, oral MPA is not a readily available medication in Brazil. Finally, our group has been publishing about PPOS cycles using dydrogesterone for a few years (Antunes *et al*., 2022; Souza *et al*., 2020; 2021).

Even though PPOS cycles are an established reality in reproductive medicine, more studies about switches in pituitary suppression therapy are needed. Therefore, with this case report, we hope to present the scientific community with a new possible strategy for patients who want to try a fresh embryo transfer and present with sings of OHSS during stimulation. The change from a GnRH antagonist to dydrogesterone seems to be effective, friendlier, and more affordable than to keep a GnRH antagonist until the end of the stimulation.
